# Metabolic Syndrome and Vascular-Associated Cognitive Impairment: a Focus on Preclinical Investigations

**DOI:** 10.1007/s11892-022-01475-y

**Published:** 2022-06-23

**Authors:** Trisha A. Jenkins

**Affiliations:** Human Biosciences, School of Health and Biomedical Sciences, STEM College, RMIT University, Plenty Road, Bundoora, VIC 3083 Australia

**Keywords:** Vascular cognitive impairment, Vascular dementia, Cognition, Hippocampus, Prefrontal cortex, Preclinical models

## Abstract

**Purpose of Review:**

Metabolic syndrome is associated with an increased risk of vascular cognitive impairment or, in the more extreme, vascular dementia. Animal models are used to investigate the relationship between pathology and behaviour. This review summarizes the latest understanding of the role of the hippocampus and prefrontal cortex in vascular cognitive impairment, the influence of inflammation in this association while also commenting on some of the latest interventions proposed.

**Recent Findings:**

Models of vascular cognitive impairment and vascular dementia, whether they develop from an infarct or non-infarct base, demonstrate increased neuroinflammation, reduced neuronal function and deficits in prefrontal and hippocampal-associated cognitive domains. Promising new research shows agents and environmental interventions that inhibit central oxidative stress and inflammation can reverse both pathology and cognitive dysfunction.

**Summary:**

While preclinical studies suggest that reversal of deficits in vascular cognitive impairment models is possible, replication in patients still needs to be demonstrated.

## Introduction

Metabolic syndrome (MetSyn) is a cluster of metabolic disturbances—abdominal obesity, hypertension, glucose intolerance and atherogenic dyslipidemia (increased plasma triglycerides and decreased high-density lipoprotein cholesterol concentration)—which is associated with increased risk for heart disease, type 2 diabetes (T2D) and stroke [1, 2]. Current evidence suggests that 20–45% of the population worldwide suffer from MetSyn [3] and while the prevalence is highest among people aged over 60 [4], recent studies show that its incidence is increasing in younger age groups [5] and across all populations [6–8].

The association between MetSyn and cardiovascular risk is well studied; however, the identification of how MetSyn metabolic changes can lead to functionally debilitating changes in cognition is less understood. Vascular cognitive impairment (VCI) is a form of cognitive deficit caused by vascular abnormalities [9]. The most severe form is vascular dementia (VD), which refers to a subgroup of patients who have dementia that is largely attributable to cerebrovascular pathology, often estimated to be ~ 15–30% of dementia cases [10]. Studies demonstrate that patients with VCI display deficits in multiple cognitive domains including memory, executive functioning, processing speed and overall intellectual functioning [11–13] which can be captured under the terminology of higher order function. Anhedonia, apathy, anxiety and depression are also frequently observed in patients with VCI [14, 15].

There are currently no medications that successfully treat VCI. Interventions such as antihypertensives and statins focus on managing risk factors [16] while both these classes of medications as well as the medications used to treat Alzheimer’s disease have little to no effect on reducing or slowing cognitive deficits [17]. Given the lack of effective pharmaceuticals, progress in our understanding of the pathophysiological mechanisms involved in VCI and VD is crucial for the development of new strategies around protection and treatment.

Pathological analysis of brain changes in VCI and VD includes both neuroimaging and pathology-confirmed diagnosis [9]. Cerebrovascular changes such as lesions, microinfarcts and arteriolosclerosis are observed along with white matter hyperintensities representing white matter degeneration [18, 19]. At a cellular level, we recognize that atypical neuroinflammation and cell death are observed with levels of inflammatory markers altered in both plasma and cerebrospinal fluid [20]. Inflammatory mechanisms disrupt cerebrovascular integrity via glial activation and increased pro-inflammatory interleukins (ILs) and tumour necrosis factor (TNF)-α production, inducing vascular tissue injury and neurodegeneration [21], and centrally endothelial and neuronal cell damage [21].

The hippocampus and prefrontal cortex play an essential role in cognitive functioning. The hippocampus is important in spatial memory and episodic memory, information formation and processing and associated behavioural regulation [22], while the prefrontal cortex plays a central role in executive functioning including attention, planning, decision-making, perception and processing [23]. Patients with VD show hippocampal atrophy [24] which is attributed to loss of neurons [25] and reduction in cerebral microvasculature [26]. Post-mortem studies show IL-1β [27] and TNF-α expression [28] in the hippocampus in VD patients is significantly higher than in age-matched controls demonstrating an influence of inflammation. However, in the prefrontal cortex in VCI and VD, white matter degeneration [29] linked to neuronal dysfunction and degeneration [30] has been shown, but there are few reports of changes in inflammatory markers in this region. It is unclear if no changes have been found or if the target of the hippocampus as the ‘memory centre’ as the prime region of investigation has reduced investigation into other brain regions.

From our understanding of cognition and cognitive deficits in its many forms, it is clear that a ‘one area fits all’ concept cannot address why cognitive deficits occur after metabolic syndrome and its associated diseases. The use of preclinical models with controlled parameters is advantageous to mimic certain aspects of MetSyn and VCI in animals and explore the relationship between brain pathology and the cognitive deficits associated with the disorders.

## Models of Vascular Cognitive Impairment

VCI animal models are largely based on modelling the cerebrovascular pathology observed in patients [31] (Table 1). Transient or permanent middle cerebral artery occlusion (MCAO) or occlusion of the common carotid arteries (CCAO) are routinely used as models of stroke and VCI to induce cerebral hypoperfusion, infarction, hypoxia and hypoperfusion of white matter due to inadequate blood supply [32–34]. This is accompanied by damaged white matter with a proliferation of astrocytes and activated microglia, disintegration of white matter tracts and a reduction in myelin [35]. Alternatively, the stroke-prone spontaneously hypertensive rat (SHR/SP) is a non-surgical VCI model. This model exhibits brain lesions predominantly within the cerebral cortex and chronic vessel changes [36, 37] leading to cortical degeneration [38].

**Table 1:** Summary table of most used VCI preclinical models

Preclinial models	Performed by	Pathology	References
Middle cerebral artery occlusion (MCAO)	Transient or permanent middle cerebral artery occlusion	Hypoperfusion, infarction, cerebral ischemia, neuronal lesions, damaged white matter tracts	[19, 32]
Occlusion of the common carotid arteries (CCAO)	Transient or permanent bilateral common carotid artery occlusion	Hypoperfusion, infarction, cerebral ischemia, neuronal lesions, damaged white matter tracts	[87]
Stroke-prone spontaneously hypertensive rat (SHR/SP)	Established from a sub-strain of spontaneously hypertensive rats	Hypertension with progressive blood pressure increase during young adulthood, wall thickening in small arteries Microvascular dysfunction, blood–brain barrier breakdown, cerebrovascular lesions, hypoxia, hypoperfusion, white matter damage	[36, 37, 88]

Metabolic disturbance can be used to model systemic cause or as an adjunct to the aforementioned models with a number of rodent models that encompass features of MetSyn. Some models are inbred strains selected for one or more traits underlying MetSyn—most commonly the SHR (spontaneous hypertensive rat) modelling hypertension. Others are population models with genetic risk for MetSyn traits, such as Zucker rats which model obesity and *db/db* mice which are a model of T2D. A third group are MetSyn traits induced by environmental stressors such as being fed high-fat and/or sugar diet [39] or administration of *streptozotocin* (*STZ*), a widely used chemical for the induction of experimental diabetes in rodents [40].

With regard to neuroinflammation, both infarct and non-infarct models of VCI demonstrate upregulation of inflammatory cells and markers. Astrocytes and microglia are activated in the brain [41] and pro-inflammatory cytokines such as IL-1β, IL-6 and TNF-α are increased [42, 43]. Inflammatory mediators such as matrix metalloproteinases are also upregulated [44].

Cognitive tests in VCI models demonstrate impaired memory. Spatial reference memory and working memory is deficient in VCI models [45, 46] which is influenced largely by the hippocampus. In this review, we will focus on the latest findings concerning hippocampal and prefrontal cortex pathology and cognitive deficits in VCI models, and endeavour to assess the plethora of new generation treatments proposed to reverse these.

## Hippocampal and Prefrontal Pathology in VCI Models

Carotid artery occlusion and MCAO models produce a loss of hippocampal neurons [47], impaired spatial memory [48–50], elevated hippocampal levels of TNF-α and IL-6, as well as increased apoptotic cell death in the hippocampus [50]. Much less is known about changes in prefrontal cortices in preclinical VCI and VD models; however, a recent study has demonstrated that two-vessel carotid artery occlusion (2-VO) reduced learning in a novel object recognition prefrontal-dependant test and lowered expression of synaptic markers in the prefrontal cortex [51•].

Utilizing a STZ/MCAO MetSyn-VCI model, researchers observed that diabetes induced hippocampal-dependant impaired cognitive function in the Y-maze, social recognition and novel object recognition tasks in concert with upregulation of NLRP3 (NOD-, LRR- and pyrin domain-containing protein 3) inflammasome expression in the hippocampus [52•]. These effects are further increased by MCAO stroke. This hippocampal inflammatory response is accompanied by higher levels of hippocampal cell death, vascular remodelling and greater astrocyte reactivity [52•]. Increased NLRP3 has also been observed in the hippocampus of the CCAO model of VCI [53], along with shrinkage, disorganization and loss of hippocampal neurons [54] and an impairment in spatial memory as observed by the Morris water maze task [54, 55] and radial arm maze [56]. Protein and mRNA levels of toll-like receptor 4 (TLR4) were increased after CCAO in microglia and neurons of the hippocampus [57] and the downstream inflammatory cytokines (IL-6) and TNF*-α* [57] while microvessels were observed to be shorter and fragmented [56]*.*

Looking at non-surgical models, a decrease in neuron number and vitality is observed in the frontal cortex and hippocampus of Zucker rats, along with a reduction in synaptic markers and a memory retention deficit in the passive avoidance task [58, 59]. Conversely in the *db/db* model hippocampal long-term potentiation is inhibited and memory impaired [60, 61] while (like surgical VCI models) inflammation and neuronal pathology is increased in the hippocampus and prefrontal cortex [61, 62]. High-fat diet-induced obesity also causes cognition impairment, downregulation of neuroplasticity-associated proteins and increases in inflammation including astrocytic reactivity in the hippocampus and prefrontal cortex [63–65].

Based on these recent studies models of VCI and VD, whether they develop from a surgical or metabolic base, deficits in prefrontal and hippocampal-associated cognitive domains, decreased neuronal function and increased neuroinflammation are observed. However, we are little closer to identifying the cellular and molecular mechanisms underlying VCI and VD. Working backwards from interventions that show attenuation of deficits is providing a potential alternative path to identifying potential targets for treatment of VCI and VD.

## New Generation Treatments and Environmental Interventions

Several new treatments have been proposed to reverse the cognitive and pathological deficits in animal models of VCI. These include anti-inflammatory, antihypertensive or antioxidant pharmacological interventions that may guide researchers towards appropriate mechanisms of action for human treatments. Other potential interventions are environmental, where the mechanisms are often poorly understood.

Injection of MCC950, a selective inhibitor of the NLRP3 inflammasome, after reperfusion in the STZ/MCAO MetSyn-VCI model ameliorated the diabetes-mediated deficits in hippocampal-dependant memory, lowered cell death of the neurons in the CA1 and dentate gyrus regions of the hippocampus and reduced levels of IL-1β and NLRP3 after MCAO [52•], suggesting that NLRP3 is a potential therapeutic target to treat cognitive impairment. In another study, osthole, a coumarin Chinese herb compound and inhibitor of NLRP3 protein expression, attenuated cognitive dysfunction in a VCI rat model induced by CCAO, evidenced by reversing spatial and working deficits, and inhibiting microglia activation in the hippocampus [66]. This notion is further strengthened by a recent study from Du and colleagues where acupuncture treatment reduced cognitive decline and hippocampal neuronal death in a model of VCI induced by CCAO by decreasing NLRP3 inflammasome and IL-1β expression in the hippocampus [53]. The molecular mechanisms of acupuncture treatment in this model are suggested to be via inhibition of thioredoxin-interacting protein (TXNIP) which plays a vital role in NLRP3 inflammasome activation, with TXNIP small interfering RNA (siRNA) producing similar effects as acupuncture on memory and hippocampal neuron survival in the CCAO VCI model [53, 67]. In a further 2-VO study, acupuncture treatment reduced the levels of inflammatory cytokines in the hippocampus which was associated with lowered expression of TLR4 in the microglia, but not neurons, of the hippocampus [57]. A TLR4 antagonist, TAK-242, had similar effects as acupuncture on inflammation in these rats, while the TLR4 agonist lipopolysaccharide inhibited the beneficial effects of acupuncture [57]. In patients, inflammasome activation and cytokine production is related to the risk factors of VCI. A 2022 article by Poh and colleagues summarize these effects [68•] and report that therapeutic agents targeting IL-1β and IL-18 are currently undergoing investigation in clinical trials and showing positive results in patient outcomes [68•, 69].

Chronic 3-month treatment with the antihypertensive amlodipine, a voltage-dependent Ca^2+^-channel blocker in BPH/2 mice prevented hypertension-related damage to functional hyperemia through the diminished activity of the capillary endothelial cell inward-rectifier potassium channel, Kir2.1 [70]. Interestingly, this result was not reproduced with the antihypertensive losartan, an angiotensin II receptor antagonist [70], aligning with clinical findings which demonstrate that calcium-channel blockers provide greater cerebrovascular protection than angiotensin-related antihypertensives in the prevention of dementia [71].

Recent studies suggested that antioxidants also have promising effects in VCI and VD models. The Chinese herb mix Chitosan reduces hippocampal-memory impairment and reduces neuron cell loss induced by CCAO while also significantly reversing reactive oxygen species production, neuronal apoptosis and microglia overactivation in the hippocampus through activation of the antioxidative response element Nrf2 (NF-E2 p45-related factor 2) causing the subsequent upregulation in the expression of many cytoprotective enzymes [72]. Meanwhile, Pinocembrin, a flavonoid with antioxidant properties [73] alleviated learning and memory deficits induced by CCAO and attenuated hippocampal neuronal damage by inducing the upregulation of the Reelin-Dab1 signalling pathway in the hippocampus [74], previously reported to exert a key role in the adult brain by promoting learning and memory [75]. Moreover in a dietary MetSyn-VCI model, gallic acid, a polyphenol and antioxidant present in grapes and green tea [76] improved recognition memory and increased hippocampal dendritic spine numbers by reducing the oxidative and inflammatory environment [77].

Evidence suggests that there are effects of physical exercise on neuroplasticity, learning and memory and investigations in preclinical models of MetSyn-VCI have produced very positive results [78, 79]. In CCAO rats modelling vascular dementia, exercise has demonstrated improvement in passive avoidance memory [80] and novel object recognition memory [51•] and increases in synaptic plasticity markers in the hippocampus and prefrontal cortex [51•, 81]. Environmental enrichment, a combination of voluntary exercise with stimulated surroundings and social interaction, alleviates memory impairment [82, 83] induced by CCAO and attenuates astrocyte activation and increases microvessel length in the hippocampus [82, 83]. Transcranial direct current stimulation (tCDS) has also been shown to stimulate increases in neuroplasticity and hippocampal long-term potentiation [84], the molecular basis of memory [85]. In a CCAO rat model, tDCS significantly alleviated the decreased hippocampal protein levels of IL-1β, IL-6 and TNF-α and memory impairment observed in the Morris water maze [54].

## Conclusion

Using models of MetSyn, VCI and VD researchers show cognitive and pathological deficits that model what is observed in humans. Promising reversal of these deficits is observed with environmental interventions, anti-inflammatory agents and antioxidants, although whether this can be replicated in patients is still to be seen. What is evident from the latest studies is that an expansion of focus is required. Interestingly, Ward and colleagues suggest that when investigating the hippocampus’s role in VCI, we should consider it as a conceptual neurovascular unit which is composed of neurons, endothelial cells and glial cells, assessing the interaction between vasculature and the surrounding brain cells rather than neurons alone [52•]. This concept is repeated by Smith et al. [86] when we advance to clinical trials who suggest that future clinical trials should investigate the broad range of interventions in preclinical models including oxidative stress and inflammation pathobiology to target the neurovascular unit in patients.

## Abbreviations

2-VO: Two-vessel carotid artery occlusion
CCAO: Occlusion of the common carotid arteries
ILs: Interleukins
MCAO: Middle cerebral artery occlusion
MetSyn: Metabolic syndrome
NLRP3: NOD-, LRR- and pyrin domain-containing protein 3
Nrf2: NF-E2 p45-related factor 2
siRNA: Small interfering RNA
SHR/SP: Stroke-prone spontaneously hypertensive rat
STZ: Streptozotocin
tCDS: Transcranial direct current stimulation
TLR-4: Toll-like receptor 4
TNF: Tumour necrosis factor
TXNIP: Thioredoxin-interacting protein
VCI: Vascular cognitive impairment
VD: Vascular dementia

## References

[1] Saklayen MG (2018). The global epidemic of the metabolic syndrome. Curr Hypertens Rep.

[2] Alberti KG, Eckel RH, Grundy SM, Zimmet PZ, Cleeman JI, Donato KA (2009). Harmonizing the metabolic syndrome: a joint interim statement of the International Diabetes Federation Task Force on Epidemiology and Prevention; National Heart, Lung, and Blood Institute; American Heart Association; World Heart Federation; International Atherosclerosis Society; and International Association for the Study of Obesity. Circulation.

[3] Engin A (2017). The definition and prevalence of obesity and metabolic syndrome. Adv Exp Med Biol.

[4] Ravaglia G, Forti P, Maioli F, Bastagli L, Chiappelli M, Montesi F (2006). Metabolic syndrome: prevalence and prediction of mortality in elderly individuals. Diabetes Care.

[5] Poyrazoglu S, Bas F, Darendeliler F (2014). Metabolic syndrome in young people. Curr Opin Endocrinol Diabetes Obes.

[6] Ranasinghe P, Mathangasinghe Y, Jayawardena R, Hills AP, Misra A. Prevalence and trends of metabolic syndrome among adults in the asia-pacific region: a systematic review. Bmc Public Health. 2017;17. ARTN 101 10.1186/s12889-017-4041-1. 10.1186/s12889-017-4041-1 PMC5251315 28109251

[7] Sherling DH, Perumareddi P, Hennekens CH (2017). Metabolic syndrome: clinical and policy implications of the new silent killer. J Cardiovasc Pharm T.

[8] Scuteri A, Laurent S, Cucca F, Cockcroft J, Cunha PG, Manas L (2015). Metabolic syndrome across Europe: different clusters of risk factors. Eur J Prev Cardiol.

[9] van der Flier WM, Skoog I, Schneider JA, Pantoni L, Mok V, Chen CLH (2018). Vascular cognitive impairment. Nat Rev Dis Primers.

[10] Goodman RA, Lochner KA, Thambisetty M, Wingo TS, Posner SF, Ling SM (2017). Prevalence of dementia subtypes in United States Medicare fee-for-service beneficiaries, 2011–2013. Alzheimers Dement.

[11] Bokura H, Nagai A, Oguro H, Kobayashi S, Yamaguchi S (2010). The association of metabolic syndrome with executive dysfunction independent of subclinical ischemic brain lesions in Japanese adults. Dement Geriatr Cogn Disord.

[12] Muller M, van Raamt F, Visseren FL, Kalmijn S, Geerlings MI, Mali WP (2010). Metabolic syndrome and cognition in patients with manifest atherosclerotic disease: the SMART study. Neuroepidemiology.

[13] Yates KF, Sweat V, Yau PL, Turchiano MM, Convit A (2012). Impact of metabolic syndrome on cognition and brain: a selected review of the literature. Arterioscler Thromb Vasc Biol.

[14] Gulpers B, Ramakers I, Hamel R, Kohler S, Oude Voshaar RC, Verhey F (2016). Anxiety as a predictor for cognitive decline and dementia: a systematic review and meta-analysis. Am J Geriat Psychiat.

[15] Diniz BS, Butters MA, Albert SM, Dew MA, Reynolds CF (2013). Late-life depression and risk of vascular dementia and Alzheimer’s disease: systematic review and meta-analysis of community-based cohort studies. Brit J Psychiat.

[16] Sinha K, Sun C, Kamari R, Bettermann K (2020). Current status and future prospects of pathophysiology-based neuroprotective drugs for the treatment of vascular dementia. Drug Discov Today.

[17] Appleton JP, Scutt P, Sprigg N, Bath PM (2017). Hypercholesterolaemia and vascular dementia. Clin Sci (Lond).

[18] Ihara M, Polvikoski TM, Hall R, Slade JY, Perry RH, Oakley AE (2010). Quantification of myelin loss in frontal lobe white matter in vascular dementia, Alzheimer’s disease, and dementia with Lewy bodies. Acta Neuropathol.

[19] Alber J, Alladi S, Bae HJ, Barton DA, Beckett LA, Bell JM (2019). White matter hyperintensities in vascular contributions to cognitive impairment and dementia (VCID): Knowledge gaps and opportunities. Alzheimers Dement (N Y).

[20] Metti AL, Cauley JA (2012). How predictive of dementia are peripheral inflammatory markers in the elderly?. Neurodegener Dis Manag.

[21] Wang X, Zhang B, Xia R, Jia Q (2020). Inflammation, apoptosis and autophagy as critical players in vascular dementia. Eur Rev Med Pharmacol Sci.

[22] Bales KL, Lewis-Reese AD, Pfeifer LA, Kramer KM, Carter CS (2007). Early experience affects the traits of monogamy in a sexually dimorphic manner. Dev Psychobiol.

[23] Miller EK, Cohen JD (2001). An integrative theory of prefrontal cortex function. Annu Rev Neurosci.

[24] van de Pol L, Gertz HJ, Scheltens P, Wolf H (2011). Hippocampal atrophy in subcortical vascular dementia. Neurodegener Dis.

[25] Vijayakumar A, Vijayakumar A (2013). Comparison of hippocampal volume in dementia subtypes. ISRN Radiol.

[26] Kril JJ, Patel S, Harding AJ, Halliday GM (2002). Patients with vascular dementia due to microvascular pathology have significant hippocampal neuronal loss. J Neurol Neurosurg Psychiatry.

[27] Cacabelos R, Alvarez XA, Fernandez-Novoa L, Franco A, Mangues R, Pellicer A (1994). Brain interleukin-1 beta in Alzheimer’s disease and vascular dementia. Methods Find Exp Clin Pharmacol.

[28] Belkhelfa M, Beder N, Mouhoub D, Amri M, Hayet R, Tighilt N (2018). The involvement of neuroinflammation and necroptosis in the hippocampus during vascular dementia. J Neuroimmunol.

[29] Craggs LJ, Yamamoto Y, Ihara M, Fenwick R, Burke M, Oakley AE (2014). White matter pathology and disconnection in the frontal lobe in cerebral autosomal dominant arteriopathy with subcortical infarcts and leukoencephalopathy (CADASIL). Neuropathol Appl Neurobiol.

[30] Foster V, Oakley AE, Slade JY, Hall R, Polvikoski TM, Burke M (2014). Pyramidal neurons of the prefrontal cortex in post-stroke, vascular and other ageing-related dementias. Brain.

[31] Hort J, Valis M, Kuca K, Angelucci F (2019). Vascular cognitive impairment: information from animal models on the pathogenic mechanisms of cognitive deficits. Int J Mol Sci.

[32] Fluri F, Schuhmann MK, Kleinschnitz C (2015). Animal models of ischemic stroke and their application in clinical research. Drug Des Dev Ther.

[33] Duncombe J, Kitamura A, Hase Y, Ihara M, Kalaria RN, Horsburgh K (2017). Chronic cerebral hypoperfusion: a key mechanism leading to vascular cognitive impairment and dementia. Closing the translational gap between rodent models and human vascular cognitive impairment and dementia. Clin Sci.

[34] Yang Y, Kimura-Ohba S, Thompson J, Rosenberg GA (2016). Rodent models of vascular cognitive impairment. Transl Stroke Res.

[35] Jalal FY, Yang Y, Thompson J, Lopez AC, Rosenberg GA (2012). Myelin loss associated with neuroinflammation in hypertensive rats. Stroke.

[36] Kimura S, Saito H, Minami M, Togashi H, Nakamura N, Nemoto M (2000). Pathogenesis of vascular dementia in stroke-prone spontaneously hypertensive rats. Toxicology.

[37] Saito H, Togashi H, Yoshioka M, Nakamura N, Minami M, Parvez H (1995). Animal models of vascular dementia with emphasis on stroke-prone spontaneously hypertensive rats. Clin Exp Pharmacol Physiol Suppl.

[38] Yamaguchi M, Sugimachi K, Nakano K, Fujimoto M, Takahashi M, Chikugo T (1994). Memory deficit accompanying cerebral neurodegeneration after stroke in stroke-prone spontaneously hypertensive rats (SHRSP). Acta Neurochir Suppl (Wien).

[39] Kwitek AE (2019). Rat models of metabolic syndrome. Methods Mol Biol.

[40] Szkudelski T (2001). The mechanism of alloxan and streptozotocin action in B cells of the rat pancreas. Physiol Res.

[41] Farkas E, Luiten PG, Bari F (2007). Permanent, bilateral common carotid artery occlusion in the rat: a model for chronic cerebral hypoperfusion-related neurodegenerative diseases. Brain Res Rev.

[42] Fu X, Zhang J, Guo L, Xu Y, Sun L, Wang S (2014). Protective role of luteolin against cognitive dysfunction induced by chronic cerebral hypoperfusion in rats. Pharmacol Biochem Behav.

[43] Kim MS, Bang JH, Lee J, Han JS, Kang HW, Jeon WK (2016). Fructus mume ethanol extract prevents inflammation and normalizes the septohippocampal cholinergic system in a rat model of chronic cerebral hypoperfusion. J Med Food.

[44] Ueno M, Wu B, Nishiyama A, Huang CL, Hosomi N, Kusaka T (2009). The expression of matrix metalloproteinase-13 is increased in vessels with blood-brain barrier impairment in a stroke-prone hypertensive model. Hypertens Res.

[45] Jiwa NS, Garrard P, Hainsworth AH (2010). Experimental models of vascular dementia and vascular cognitive impairment: a systematic review. J Neurochem.

[46] Pappas BA, de la Torre JC, Davidson CM, Keyes MT, Fortin T (1996). Chronic reduction of cerebral blood flow in the adult rat: late-emerging CA1 cell loss and memory dysfunction. Brain Res.

[47] Lee TK, Kim H, Song M, Lee JC, Park JH, Ahn JH (2019). Time-course pattern of neuronal loss and gliosis in gerbil hippocampi following mild, severe, or lethal transient global cerebral ischemia. Neural Regen Res.

[48] Kitamura A, Saito S, Maki T, Oishi N, Ayaki T, Hattori Y (2016). Gradual cerebral hypoperfusion in spontaneously hypertensive rats induces slowly evolving white matter abnormalities and impairs working memory. J Cereb Blood Flow Metab.

[49] Tuo QZ, Lei P, Jackman KA, Li XL, Xiong H, Li XL (2017). Tau-mediated iron export prevents ferroptotic damage after ischemic stroke. Mol Psychiatry.

[50] Khoshnam SE, Sarkaki A, Rashno M, Farbood Y (2018). Memory deficits and hippocampal inflammation in cerebral hypoperfusion and reperfusion in male rats: neuroprotective role of vanillic acid. Life Sci.

[51.•] Dong J, Zhao J, Lin Y, Liang H, He X, Zheng X (2018). Exercise improves recognition memory and synaptic plasticity in the prefrontal cortex for rats modelling vascular dementia. Neurol Res.

[52.•] Ward R, Li W, Abdul Y, Jackson L, Dong G, Jamil S (2019). NLRP3 inflammasome inhibition with MCC950 improves diabetes-mediated cognitive impairment and vasoneuronal remodeling after ischemia. Pharmacol Res.

[53] Du SQ, Wang XR, Zhu W, Ye Y, Yang JW, Ma SM (2018). Acupuncture inhibits TXNIP-associated oxidative stress and inflammation to attenuate cognitive impairment in vascular dementia rats. CNS Neurosci Ther.

[54] Guo T, Fang J, Tong ZY, He S, Luo Y (2020). Transcranial direct current stimulation ameliorates cognitive impairment via modulating oxidative stress, inflammation, and autophagy in a rat model of vascular dementia. Front Neurosci.

[55] Moghaddasi M, Taati M, Asadian P, Khalatbary AR, Asaei R, Pajouhi N (2017). The effects of two-stage carotid occlusion on spatial memory and pro-inflammatory markers in the hippocampus of rats. J Physiol Sci.

[56] Lee JM, Lee JH, Song MK, Kim YJ (2021). NXP031 improves cognitive impairment in a chronic cerebral hypoperfusion-induced vascular dementia rat model through Nrf2 signaling. Int J Mol Sci.

[57] Wang L, Yang JW, Lin LT, Huang J, Wang XR, Su XT (2020). Acupuncture attenuates inflammation in microglia of vascular dementia rats by inhibiting miR-93-mediated TLR4/MyD88/NF-kappaB signaling pathway. Oxid Med Cell Longev.

[58] Tomassoni D, Martinelli I, Moruzzi M, Micioni Di Bonaventura MV, Cifani C, Amenta F (2020). Obesity and age-related changes in the brain of the Zucker Lepr (fa/fa) rats. Nutrients..

[59] Martinelli I, Tomassoni D, Roy P, Amenta F, Tayebati SK (2021). Altered brain cholinergic and synaptic markers in obese Zucker rats. Cells.

[60] Zhang SY, Ji SX, Bai XM, Yuan F, Zhang LH, Li J (2019). L-3-n-butylphthalide attenuates cognitive deficits in db/db diabetic mice. Metab Brain Dis.

[61] Ye T, Meng X, Wang R, Zhang C, He S, Sun G (2018). Gastrodin alleviates cognitive dysfunction and depressive-like behaviors by inhibiting ER stress and NLRP3 inflammasome activation in db/db mice. Int J Mol Sci.

[62] Yermakov LM, Drouet DE, Griggs RB, Elased KM, Susuki K (2018). Type 2 diabetes leads to axon initial segment shortening in db/db mice. Front Cell Neurosci.

[63] Li F, Liu BB, Cai M, Li JJ, Lou SJ (2018). Excessive endoplasmic reticulum stress and decreased neuroplasticity-associated proteins in prefrontal cortex of obese rats and the regulatory effects of aerobic exercise. Brain Res Bull.

[64] Spencer SJ, D’Angelo H, Soch A, Watkins LR, Maier SF, Barrientos RM (2017). High-fat diet and aging interact to produce neuroinflammation and impair hippocampal- and amygdalar-dependent memory. Neurobiol Aging.

[65] Bondan EF, Cardoso CV, Martins MDM, Otton R (2019). Memory impairments and increased GFAP expression in hippocampal astrocytes following hypercaloric diet in rats. Arq Neuro-Psiquiat.

[66] Liu Y, Chen X, Gong Q, Shi J, Li F (2020). Osthole improves cognitive function of vascular dementia rats: reducing Abeta deposition via inhibition NLRP3 inflammasome. Biol Pharm Bull.

[67] Zhou R, Tardivel A, Thorens B, Choi I, Tschopp J (2010). Thioredoxin-interacting protein links oxidative stress to inflammasome activation. Nat Immunol.

[68.•] Poh L, Sim WL, Jo DG, Dinh QN, Drummond GR, Sobey CG, et al. 2022 The role of inflammasomes in vascular cognitive impairment. Mol Neurodegener. 2022;17(1). ARTN 4 10.1186/s13024-021-00506-8. A **2022 review into the molecular and cellular mechanisms involved in the pathogenesis of inflammasome signaling in VCI. Includes information on animal models and clinical studies.** 10.1186/s13024-021-00506-8 PMC8744307 35000611

[69] Emsley HCA, Smith CJ, Georgiou RF, Vail A, Hopkins SJ, Rothwell NJ (2005). A randomised phase II study of interleukin-1 receptor antagonist in acute stroke patients. J Neurol Neurosur Ps.

[70] Koide M, Harraz OF, Dabertrand F, Longden TA, Ferris HR, Wellman GC (2021). Differential restoration of functional hyperemia by antihypertensive drug classes in hypertension-related cerebral small vessel disease. J Clin Invest.

[71] Staessen JA, Thijs L, Richart T, Odili AN, Birkenhager WH (2011). Placebo-controlled trials of blood pressure-lowering therapies for primary prevention of dementia. Hypertension.

[72] Jiang P, Chen L, Sun J, Li J, Xu J, Liu W (2019). Chotosan ameliorates cognitive impairment and hippocampus neuronal loss in experimental vascular dementia via activating the Nrf2-mediated antioxidant pathway. J Pharmacol Sci.

[73] Liang J, Yu Y, Wang B, Lu B, Zhang J, Zhang H (2013). Ginsenoside Rb1 attenuates oxygen-glucose deprivation-induced apoptosis in SH-SY5Y cells via protection of mitochondria and inhibition of AIF and cytochrome c release. Molecules.

[74] Kang ZC, Wang HG, Yang YL, Zhao XY, Zhou QM, Yang YL (2020). Pinocembrin ameliorates cognitive impairment induced by vascular dementia: contribution of Reelin-dab1 signaling pathway. Drug Des Devel Ther.

[75] Wasser CR, Herz J (2017). Reelin: Neurodevelopmental architect and homeostatic regulator of excitatory synapses. J Biol Chem.

[76] Fernandes FHA, Salgado HRN (2016). Gallic Acid: Review of the methods of determination and quantification. Crit Rev Anal Chem.

[77] Aamodt K, Abelev B, Quintana AA, Adamova D, Adare AM, Aggarwal MM (2010). Elliptic flow of charged particles in Pb-Pb collisions at sqrt[S(NN)] = 2.76 TeV. Phys Rev Lett.

[78] Nguyen JC, Killcross AS, Jenkins TA (2013). Effect of low-intensity treadmill exercise on behavioural measures and hippocampal parvalbumin immunoreactivity in the rat. Behav Brain Res.

[79] Nithianantharajah J, Hannan AJ (2006). Enriched environments, experience-dependent plasticity and disorders of the nervous system. Nature reviewsNeuroscience.

[80] Zhang L, Fan Y, Kong X, Hao W (2020). Neuroprotective effect of different physical exercises on cognition and behavior function by dopamine and 5-HT level in rats of vascular dementia. Behav Brain Res.

[81] Lin YY, Dong JT, Yan TB, He XK, Zheng XY, Liang HY (2015). Involuntary, forced and voluntary exercises are equally capable of inducing hippocampal plasticity and the recovery of cognitive function after stroke. Neurol Res.

[82] Song MK, Kim YJ, Lee JM, Kim YJ (2020). Neurovascular integrative effects of long-term environmental enrichment on chronic cerebral hypoperfusion rat model. Brain Res Bull.

[83] Park JM, Seong HH, Jin HB, Kim YJ (2017). The effect of long-term environmental enrichment in chronic cerebral hypoperfusion-induced memory impairment in rats. Biol Res Nurs.

[84] Ranieri F, Podda MV, Riccardi E, Frisullo G, Dileone M, Profice P (2012). Modulation of LTP at rat hippocampal CA3-CA1 synapses by direct current stimulation. J Neurophysiol.

[85] Abraham WC, Jones OD, Glanzman DL (2019). Is plasticity of synapses the mechanism of long-term memory storage?. NPJ Sci Learn.

[86] Smith EE, Cieslak A, Barber P, Chen J, Chen YW, Donnini I (2017). Therapeutic strategies and drug development for vascular cognitive impairment. J Am Heart Assoc.

[87] Cao DD, Bai YF, Li L (2018). Common carotid arteries occlusion surgery in adult rats as a model of chronic cerebral hypoperfusion. Bio Protocol..

[88] Nabika T, Ohara H, Kato N, Isomura M (2012). The stroke-prone spontaneously hypertensive rat: still a useful model for post-GWAS genetic studies?. Hypertens Res.

